# Editorial: Intracellular ion channels in health and disease

**DOI:** 10.3389/fphar.2024.1430150

**Published:** 2024-07-02

**Authors:** Giovanni Zifarelli, Michael Pusch, Tobias Stauber

**Affiliations:** ^1^ Istituto di Biofisica, Consiglio Nazionale delle Ricerche, Genova, Italy; ^2^ Centogene GmbH, Rostock, Germany; ^3^ Institute for Molecular Medicine, MSH Medical School Hamburg, Hamburg, Germany

**Keywords:** biophyiscs, membrane transport, intracellular ion channel, intracellular transporter, chloride ion, calcium ion

Ion channels and transporters in intracellular compartments are increasingly recognized as important players in cellular physiology and in disease (Hu et al., 2024). However, investigation of intracellular proteins, particularly ion channels and transporters, is exceedingly difficult causing the field to lag behind other more established areas of cellular physiology. However, this research area has seen a rapid expansion in recent years.

Within this context, we launched the Research Topic *Intracellular ion channels in health and disease* and invited researchers to contribute to a Research Topic of state-of-the-art articles in the field of ion channels and transporters expressed in intracellular compartments. Authors could present recent advances in the field with respect to the structure, biophysical properties, regulation, physiological and pathophysiological roles, pharmacology and drugability of intracellular channels and transporters.

We have received insightful manuscripts covering diverse aspects of the topic. Picollo reviewed vesicular chloride/proton exchangers of the CLC family that predominantly localize to the endosomal-lysosomal pathway. She summarized the newest findings on their structures by cryo-electron microscopy before she described the physiological roles of individual CLCs, and diseases resulting from their dysfunction. The latter include Dent’s disease with ClC-5, various neurological disorders with ClC-3 through −7. Dysfunction of ClC-7 additionally underlies recessive or dominant osteopetrosis. Three further articles focus on different aspects of intracellular calcium regulation. Paar et al. examined cardiac septal hypertrophies from aortic valve stenosis (AVS) or hypertrophic obstructive cardiomyopathy (HOCM) with samples from patients. They found increased fibrosis and changes in abundance of proteins associated with the mitochondrial Ca^2+^ uniporter (MCU). In AVS, expression of the sarco-endoplasmic reticulum calcium ATPase (SERCA), SERCA2a, was additionally elevated. These findings suggest that impaired mitochondrial Ca^2+^ uptake may contribute to disease progression alongside cytosolic calcium changes. Romero-Sanz et al. investigated the effects of submaximal inhibition of the SERCA in a model of Parkinson’s disease induced in *C. elegans* worms by the mitochondrial complex I inhibitor rotenone. Rotenone caused various detrimental effects in the worms, including decreased lifespan, size, fertility, motility, and altered mitochondrial function and structure. These effects were partially or fully reversed by SERCA inhibition using RNA interference against *sca-1*, the SERCA orthologue in *C. elegans*. These data suggest that modulation of intracellular Ca^2+^ by targeting SERCA could be a potential therapeutic approach for preventing or treating neurodegenerative diseases like Parkinson’s. Lastly, Wang et al. reviewed different calcium-sensing receptors and channels of the plasma membrane and intracellular organelles in respect to their function in skin function maintenance and dermatological diseases. The different types of calcium channels they cover include voltage-gated calcium channels, transient receptor potential (TRP) channels, store-operated calcium channels and the ER calcium release channels, inositol trisphosphate (IP3) receptor and ryanodine receptor. Besides their functional roles, the review highlights potential applications in therapeutic approaches in various dermatological diseases.

Taken together, four articles were published in this Research Topic, which have covered various aspects of intracellular ion channels, in respect to the ion species, to organelles and to the physiological or pathological context. We believe that all selected articles published in this Research Topic have provided insights in the molecular mechanisms, physiological roles and translational impact of intracellular ion channels, with promising future perspectives. In the coming years, research on ion channels of intracellular membranes will make significant advancements, addressing existing questions and undoubtedly revealing surprising functions.
